# Participation of Endosomes in Toll-Like Receptor 3 Transportation Pathway in Murine Astrocytes

**DOI:** 10.3389/fncel.2020.544612

**Published:** 2020-11-17

**Authors:** Matylda B. Mielcarska, Karolina P. Gregorczyk-Zboroch, Lidia Szulc-Dąbrowska, Magdalena Bossowska-Nowicka, Zbigniew Wyżewski, Joanna Cymerys, Marcin Chodkowski, Paula Kiełbik, Michał M. Godlewski, Małgorzata Gieryńska, Felix N. Toka

**Affiliations:** ^1^Division of Immunology, Department of Preclinical Sciences, Institute of Veterinary Medicine, Warsaw University of Life Sciences, Warsaw, Poland; ^2^Institute of Biological Sciences, Cardinal Stefan Wyszynski University in Warsaw, Warsaw, Poland; ^3^Division of Microbiology, Department of Preclinical Sciences, Institute of Veterinary Medicine, Warsaw University of Life Sciences, Warsaw, Poland; ^4^Division of Physiology, Department of Physiological Sciences, Institute of Veterinary Medicine, Warsaw University of Life Sciences, Warsaw, Poland; ^5^Center for Integrative Mammalian Research, Department of Biomedical Sciences, Ross University School of Veterinary Medicine, Basseterre, Saint Kitts and Nevis

**Keywords:** innate immunity, TLR3, endocytic trafficking, endosomes, astrocytes

## Abstract

TLR3 provides immediate type I IFN response following entry of stimulatory PAMPs into the CNS, as it is in HSV infection. The receptor plays a vital role in astrocytes, contributing to rapid infection sensing and suppression of viral replication, precluding the spread of virus beyond neurons. The route of TLR3 mobilization culminating in the receptor activation remains unexplained. In this research, we investigated the involvement of various types of endosomes in the regulation of the TLR3 mobility in C8-D1A murine astrocyte cell line. TLR3 was transported rapidly to early EEA1-positive endosomes as well as LAMP1-lysosomes following stimulation with the poly(I:C). Later, TLR3 largely associated with late Rab7-positive endosomes. Twenty-four hours after stimulation, TLR3 co-localized with LAMP1 abundantly in lysosomes of astrocytes. TLR3 interacted with poly(I:C) intracellularly from 1 min to 8 h following cell stimulation. We detected TLR3 on the surface of astrocytes indicating constitutive expression, which increased after poly(I:C) stimulation. Our findings contribute to the understanding of cellular modulation of TLR3 trafficking. Detailed analysis of the TLR3 transportation pathway is an important component in disclosing the fate of the receptor in HSV-infected CNS and may help in the search for rationale therapeutics to control the replication of neuropathic viruses.

## Introduction

Glial cells consist of microglia, oligodendrocytes, NG-2 glia, and astrocytes, the last of which constitute the most abundant cells in the brain (Dimou and Gallo, 2015). In addition to maintaining the proper functioning and structure of the blood–brain barrier (BBB), astrocytes release a variety of neurotransmitters, provide substrates for the production of ATP by neurons, and contribute to water and ion homeostasis in the brain (Jäkel and Dimou, 2017; Vasile et al., 2017; Ludwig and Das, 2019). Furthermore, they regulate the immune response and are important mediators of inflammatory responses in the central nervous system (CNS).

Often referred to as innate immunity sentinels within the CNS (Scumpia et al., 2005), astrocytes are involved in the immune responses during diverse neurological disorders. In response to brain injury, neuroinflammation or neurodegeneration, numerous concomitant astrocytic processes lead to a condition called reactive astrogliosis (Strömberg et al., 1986). Notably, the spectrum of changes during astrogliosis significantly depends on a variety of divergent signaling events that are conditioned both by nature and severity of the CNS abnormality (Sofroniew, 2015). During CNS insult such as herpes simplex encephalitis (HSE) viral, reactive astrocytes actively participate in the inflammatory response and secrete various cytokines and chemokines, such as TNF-α, IFN-β, IL-8, and CXCL10, which significantly affect both initiation and/or progression of the inflammation in CNS (Park et al., 2006).

The role of astrocytes infection has been studied in various models to support the notion that these cells affect the location, duration and extent of the neuroinflammation in the brain (Cekanaviciute and Buckwalter, 2016). However, more careful research of neurological disease models needs to be implemented further to unravel the complexity of the astrocytic functions in the brain. In the course of viral dsRNA-mediated inflammation, IFN-β produced by astrocytes may induce anti-inflammatory responses and comprehensively regulate the activation of innate and adaptive immune cells (Park et al., 2006; Bolívar et al., 2018). During CNS disease such as multiple sclerosis (MS), therapeutically administered IFN-β alleviates excess inflammation through a pleiotropic effect on the immune system (Kasper and Reder, 2014). In light of the “cytokine storm” phenomenon, e.g., during COVID-19 infection with upraised magnitude and range of cytokines and chemokines, it is possible that pattern recognition receptors (PRRs) activate and modulate innate responses by affecting the expression of IFN-α and -β genes as well as IFN-stimulated genes. However, due to high production of pro-inflammatory cytokines during COVID-19, the anti-inflammatory effect of IFNs may be overshadowed, and in that case leading dysfunction in the CNS (Acharya et al., 2020; Yesilkaya and Balcioglu, 2020). By engaging viral dsRNA, toll-like receptor 3 (TLR3) triggers type I IFN signaling which may play a protective role in the CNS during HSV infection and balance the cytokine and chemokine production during HSE. TLR3 ligands are used as adjuvants in vaccines due to high receptor expression in human and murine dendritic cells (Perales-Linares and Navas-Martin, 2013). However, cytokine signaling pathway redundancy may also be observed with respect to TLR3 activation and data are varying regarding TLR3 role in protective immunity during other viral infections. Therefore, TLR3 contribution to astrogliosis development, cytokine production and subjugation of the inflammatory state during HSE presents an interesting topic of concern and constitutes a target for potential immunotherapies.

Herpes simplex encephalitis is the most common sporadic viral encephalitis in Western countries and accounts for 10–20% of all CNS viral infections (Whitley and Gnann, 2002). It is a severe disease of high morbidity and mortality and often leads to lifelong neurological deficiencies. HSE is caused by primary or recurrent infection with herpes simplex virus type 1 or type 2 (HSV-1 and HSV-2), which establish latency in sensory neurons belonging to the trigeminal ganglion (TG), and possibly in the CNS (Baringer and Pisani, 1994; Held and Derfuss, 2011). HSV-1 or HSV-2 enter the CNS *via* the trigeminal or olfactory tract, by hematogenous spread, or by axonal spread following peripheral viral reactivation (Gnann and Whitley, 2017). The protective response during HSV infection in the brain is dependent on the induction of interferon through Toll-like receptor 3 (TLR3) activation. However, recent discoveries show that deficiency of *TLR3* or TLR3 signaling pathway genes is associated with HSE among children and adults (Zhang et al., 2007; Lim et al., 2014; Mørk et al., 2015; Sironi et al., 2017).

Toll-like receptor 3 senses microbial double-stranded RNA (dsRNA), leading to up-regulation of the signaling cascade that results in inflammatory response in the form of cytokines and chemokines such as type I IFNs, IL-6, TNF-α, CXCL-8, and CXCL-10 (Alexopoulou et al., 2001). Successively, these molecules and type I IFNs in particular, stimulate natural killer cells, control promotion of dendritic cells (DCs) maturation and enhance CD8^+^ T cell responses (Cheng and Xu, 2010). Extracellular dsRNA molecules bind to specific uptake receptors on the cell surface and undergo endocytosis, a process mediated by Raftlin, in a clathrin-dependent manner to eventually anchor in endosomes or lysosomes and initiate immune responses (Tatematsu et al., 2018). Prior to ligand recognition, TLR3 is synthesized and resides in the endoplasmic reticulum (ER). Similar to other nucleic acid-sensing TLRs such as TLR7, TLR8, and TLR9, TLR3 requires UNC93B1 to exit the ER in vesicles coated in cytoplasmic coat protein II (COPII) complex, and to be further transported (Brinkmann et al., 2007; Kim et al., 2008; Lee et al., 2013). Newly synthesized receptor is trafficked to Golgi and localizes intracellularly in endosomes or lysosomes, where it undergoes proteolytic treatment and presumably recognizes dsRNA (Garcia-Cattaneo et al., 2012). There is a possibility that TLR3 residing in endosomes is not recruited through the Golgi apparatus, but directly from the ER (Johnsen et al., 2006). Endosomal sorting complex required for transportation-0 (ESCRT-0) has been implicated to control the endosomal pathway of TLR7 and TLR9 (Lee and Barton, 2014). Recently, our research has reported that Hepatocyte growth factor-regulated tyrosine kinase substrate (Hrs), component of the ESCRT-0, directly interacts with TLR3. Furthermore, both Hrs and Spleen tyrosine kinase (Syk), a protein that phosphorylates Hrs, affect the potency of TLR3-mediated antiviral response (Mielcarska et al., 2019). The presence of TLR3 has been confirmed in various subpopulations of endosomes marked with EEA1 and Rab5 (endosomes formed from the plasma membrane), Rab11 (recycling endosomes), Rab7 (mature endosomes), and LAMP1 (endosomes destined to fuse with lysosomes) (Qi et al., 2012). However, types of endosomes in which TLR3 appears, as well as the temporal dependency of the TLR3 presence in certain intracellular compartment following dsRNA stimulation of astrocytes remain to be elucidated. Importantly, TLR3 residence in different types of endosomes is diversified depending on the type of tissues, cells, and time that has elapsed since the receptor stimulation. It is also uncertain whether TLR3 is constitutively found in the dsRNA recognition site or whether it is transferred to endosomes to encounter nucleic acid as a consequence of cell stimulation. While this issue awaits experimental proof, it is very likely that proper distribution of TLR3 in the specific type of cell, and precise delivery of the receptor to the ligand recognition site is indispensable for the initiation of rapid and effective antiviral response.

In this work, we investigated TLR3 localization in murine astrocyte C8-D1A cell line following the addition of synthetic dsRNA, polyinosinic:polycytidylic acid [poly(I:C)] at nine or more different time intervals. Naked poly(I:C) applied to the medium of cultured astrocytes mimics HSV-1 infection, as viral dsRNA may appear in the extracellular environment of the brain following lysis of the infected cells. As previously described (Mielcarska et al., 2019), C8-D1A cells highly express TLR3, the TLR3 agonist induces activation of the inflammatory signaling and TLR3 presence was confirmed in the ER. In the present study, we applied confocal microscopy examination and confirmed the presence of TLR3 in early and late endosomes as well as lysosomes, identified by EEA1, Rab7 and LAMP1 markers, respectively. Furthermore, we examined expression levels for each of the endosomal markers, following poly(I:C) stimulation of astrocytes. Lastly, because in certain cell types TLR3 can perform its functions in both endosomes and plasma membrane, we showed TLR3 occurrence on the surface of astrocytes at rest, as well as after stimulation with poly(I:C).

## Materials and Methods

### Cell Culture

Murine astrocytes from C8-D1A cell line were obtained from ATCC (CRL-2541) and used in all experiments. Cells were maintained in DMEM with high glucose (GE Healthcare Life Sciences) and supplemented with 10% heat-inactivated FBS, 4.0 mM L-glutamine medium and 1% solution of penicillin G, streptomycin and amphotericin B (Merck KGaA), and cultured at 37°C in 5% CO_2_. Astrocytes were maintained under conditions described by Freshney (1994), detached by using trypsin-EDTA solution (0.25%, Merck KGaA) and transferred into new culture plates with fresh medium. Passaging of C8-D1A cells was performed twice per week and cells from passage 2–15 were used for the experiments.

### Stimulation of Astrocytes With TLR3 Ligand

C8-D1A cells were cultured for 24 h and subsequently treated with a TLR3 agonist, poly(I:C) high molecular weight (HMW) (10 μg/ml) (InvivoGen). Poly(I:C) HMW has significantly higher efficiency of TLR3 activation in various cell lines than low molecular weight (LMW) poly(I:C) (Zhou et al., 2013), and was previously validated for TLR3 potency in C8-D1A cells (Mielcarska et al., 2019). Unlike naked poly(I:C), transfected poly(I:C)/LyoVec stimulates cytosolic RIG-I and MDA-5 receptors and was used as a control (1 μg/ml). To control for the effect of LyoVec-mediated membrane solubilization or interactions of the vehicle with cellular compartments on the differential changes in the protein expression observed following cell stimulation with poly(I:C) instead of poly(I:C)/LyoVec, C8-D1A astrocytes were stimulated with LyoVec (1 μg/ml). Furthermore, LyoVec was used to verify contribution of the destabilization of membrane bilayers during experiments with poly(I:C)/LyoVec. LyoVec transfection efficiency was assessed by transfecting C8-D1A cells with LyoVec-GFP/SEAP complex according to the manufacturer’s instructions (InvivoGen). Fresh medium was added to the astrocytes at the same time as poly(I:C) or poly(I:C)/LyoVec or LyoVec. Poly(I:C) and poly(I:C)/LyoVec concentrations were established empirically in previous experiments. TLR3 stimulation times were selected based on the observation of the TLR3 presence in individual types of endosomes and rapidity of their formation. To examine protein expression, we selected poly(I:C) and poly(I:C)/LyoVec stimulation times from those in which we observed changes in expression, and analogous to those used for studies of the TLR3 presence in endosomes.

### Antibodies and Reagents

Mouse monoclonal antibodies purchased from the specified manufacturers were used in the experiments described below. LAMP1, Rab7 (Santa Cruz Biotechnology); dsRNA (English and Scientific Consulting, Hungary (SCICONS); TLR3, GAPDH (Thermo Fisher Scientific); rabbit polyclonal antibodies against EEA1 (Merck KGaA), TLR3 (Thermo Fisher Scientific); horse anti-mouse HRP-conjugated IgG antibody, goat anti-rabbit HRP-conjugated IgG antibody (Cell Signaling Technology), FITC-conjugated rat monoclonal IgG2a antibody against TLR3, FITC-conjugated rat monoclonal IgG2a isotype control antibody (Thermo Fisher Scientific); Red-X-conjugated donkey anti-mouse polyclonal IgG antibody, Red-X-conjugated donkey anti-rabbit polyclonal IgG antibody, FITC-conjugated donkey anti-mouse polyclonal IgG antibody, FITC-conjugated donkey anti-rabbit polyclonal IgG antibody (Jackson ImmunoResearch Europe Ltd.); Hoechst 33342 (Merck KGaA). Primary antibodies used in the immunofluorescence experiments and their dilutions are listed in Table 1.

**TABLE 1:** Primary antibodies used for immunofluorescence staining.

Antibody	Clone/ID	Isotype	Company	Dilution
TLR3	PA5-23105^a^	Polyclonal rabbit	Thermo Fisher Scientific	1:100
TLR3	40C1285.6^b^	Monoclonal mouse	Thermo Fisher Scientific	1:100
EEA1	07-1820^a^	Polyclonal rabbit	Merck KGaA	1:200
Rab7	B-3^a^	Monoclonal mouse	Santa Cruz Biotechnology	1:50
LAMP1	H4A3^a^	Monoclonal mouse	Santa Cruz Biotechnology	1:50
dsRNA	K1^b^	Monoclonal mouse	SCICONS	1:100

*^a^Manufacturer’s antibody identification. ^b^Clone.*

### Western Blot Analysis

At the indicated times or concentrations, C8-D1A cells were lysed in RIPA Lysis and Extraction Buffer or Pierce IP Lysis Buffer (for the western blot of LAMP1) supplemented with 1% protease and phosphatase inhibitor cocktail (Thermo Fisher Scientific). Total protein concentration was determined by the BCA Protein Assay Kit (Thermo Fisher Scientific). Twenty micrograms of protein were subjected to SDS-PAGE and electrophoretically transferred to PVDF membranes using the Bolt^®^ System (Thermo Fisher Scientific). Membranes were blocked in phosphate-buffered saline with Tween (PBST) containing 5% non-fat milk for 2 h at 22°C before incubation with antibodies against EEA1, Rab7, LAMP1 or GAPDH overnight at 4°C. After washing three times with Tris-buffered saline containing 0.1% Tween 20, membranes were probed with HRP-conjugated secondary antibodies for 1 h at room temperature and washed 3 times. The SuperSignal West Pico PLUS (Thermo Fisher Scientific) was used to develop, and X-ray film to visualize the protein bands. Densitometry analysis was performed using ImageJ (NIH Image, version 1.50i) software. GAPDH was used as the protein loading control and quantification was obtained by normalizing values to GAPDH.

### Fluorescent Confocal Microscopy

C8-D1A cells were seeded on 22-mm glass coverslips in 24-well plates. Astrocytes were left untreated or treated with poly(I:C) for indicated time points, fixed with 4% paraformaldehyde in PBS for 20 min and permeabilized with 0.5% Triton X-100 (Merck KGaA) in PBS for 15 min. The cells were blocked with 3% bovine serum albumin (BSA, Merck KGaA) with 0.1% Triton X-100 in PBS. Next, astrocytes were stained with antibodies against EEA1, Rab7, LAMP1 or TLR3 for 1 h and subsequently incubated with FITC- and rhodamine Red-X-conjugated secondary antibodies for 1 h in the dark. The nuclei were stained with 1 μg/ml Hoechst 33342 (Merck KGaA) for 5 min in the dark. Lastly, slides were mounted with ProLong Gold Antifade Reagent (Thermo Fisher Scientific). The cells were observed using Fluoview FV10i laser scanning confocal microscope (Olympus) under a 60× water-immersion objective. Images were captured using FV10i software (Olympus), converted to 24-bit tiff files for visualization and analyzed with ImageJ (NIH Image, version 1.50i) and/or QuickPHOTO MICRO (Promicra, Czech Republic, version 3.1) software. To confirm the results obtained in fluorescent confocal microscopy, analogous experiments were performed using confocal microscopy-independent technology, Duolink^®^ *in situ* proximity ligation assay PLA (Merck KGaA).

### Flow Cytometry

For detection of TLR3 on the cell surface, astrocytes were harvested with cell dissociation buffer (Thermo Fisher Scientific) and washed in ice-cold PBS (Thermo Fisher Scientific). Subsequently, cells were blocked by incubation with PBS supplemented with 30% heat-inactivated FBS (Merck KGaA) for 30 min at 4°C before antibody staining. Monoclonal rat anti-TLR3 antibodies (Thermo Fisher Scientific) directed against the extracellular domain of the TLR3 protein (1 μg) were added to 1 × 10^6^ cells for 30 min at 4°C in the dark. Following incubation, cells were washed and resuspended in ice-cold PBS and 20,000 events were recorded. Flow cytometric measurements were performed using the LSRFortessa flow cytometer (Becton Dickinson Biosciences) and analyzed with FACSDiva 7.0 software.

### TLR3 Antagonist Cell Treatments

To exclude contribution of the non-TLR3-mediated signaling pathways, we used TLR3 antagonist, *N*-[(3-chloro-6-fluorobenzo[b]thien-2-yl)carbonyl]-d-phenylalanine (CU CPT 4a; Tocris Bioscience). Cells were not treated, treated with poly(I:C) or pretreated with 20 μM TLR3 antagonist for 1 h before treatment with poly(I:C). Astrocytes were fixed on slides and the TLR3/endosomal protein interaction was examined. Furthermore, supernatants from cell cultures pretreated with CU CPT 4a for 1 h followed by stimulation with poly(I:C) were tested for IFN-β concentrations as detailed in the following section.

### ELISA

To evaluate IFN-β secretion, astrocytes were seeded in 24-well tissue culture plates at a density of 3 × 105 cell per well in 1 ml of normal growth medium. Following pretreatment with CU CPT 4a and 24 h stimulation with poly(I:C), the cell supernatants were harvested and stored at −80°C until analysis in ELISA assay. IFN-β was measured with IFN-β mouse ELISA kit (Thermo Fisher Scientific) according to the manufacturer’s protocol. The concentrations of IFN-β were determined by measuring optical densities (OD) in Epoch Microplate Spectrophotometer followed by data reduction in Gen5 software (BioTek Instruments, Inc., Winooski, VT, United States) based on a calibration curve.

### Detection of Protein Interaction by Duolink^®^ *in situ* PLA

The direct observation of TLR3-EEA1, TLR3-Rab7, and TLR3-LAMP1 interaction was carried out with Duolink^®^ *in situ* PLA kit (Merck KGaA) following the manufacturer’s protocol. In PLA, a pair of single-stranded DNA labeled secondary antibodies (PLA probes) enable to detect the interaction and determine cellular localization of the examined proteins following DNA circle amplification. The signal can be observed when the distance between the secondary antibodies does not exceed 40 nm. Primary antibodies used in the study are presented in Table 1. Images were captured using an Olympus BX60 fluorescence microscope and analyzed with QuickPHOTO MICRO (Promicra, Czech Republic, version 3.1) software.

### Statistical Analysis

Independent two sample Student’s *t*-test was used in intergroup comparisons of categorical variables and categorical variables were expressed as target protein/GAPDH ratio (percentages). Data are presented as mean ± standard deviation (SD) from at least three independent biological experiments unless otherwise indicated. *P* values ≤0.05 (^∗^) or ≤0.01 (^∗∗^) were considered as statistically significant. The calculations were performed using STATISTICA software (StatSoft, Poland).

## Results

### Expression of Endosomal Markers EEA1 and LAMP1 Is Altered Following Stimulation of Astrocytes With Poly(I:C)

Stimulation of TLR3 by its agonist leads to cell activation and production of proinflammatory cytokines. However, clear understanding of the cellular compartment(s) where the interaction of TLR3 and its agonist take place is not available. In the previous studies we determined the TLR3 expression level in C8-D1A cells and demonstrated that, upon poly(I:C) stimulation, TLR3 protein was upregulated (Mielcarska et al., 2019). Early and late endosomes, as well as lysosomes, may be involved in the TLR3 transportation pathway following the addition of the TLR3 ligand. The innate immune system senses foreign dsRNA in at least two different ways, the TLR3 pathway detects dsRNA in endosomes, while RIG-I and MDA5 receptors recognize dsRNA localized in the cytoplasm (Gitlin et al., 2006). Therefore, as a control we used synthetic dsRNA complexed with the transfection reagent, cationic lipid LyoVec. Unlike naked poly(I:C), transfected poly(I:C)/LyoVec is sensed only by cytosolic RIG-I and MDA5. Poly(I:C) and poly(I:C)/LyoVec were broadly used in research as the activators of dsRNA signaling pathways (Katashiba et al., 2011; Liu et al., 2012; Qian et al., 2013; Zhou et al., 2013; Ohta et al., 2014; Dauletbaev et al., 2015, 2017; Mielcarska et al., 2019; Singaravelu et al., 2019). To shed light on the possible site of TLR3 interaction with ligand, we first investigated the expression levels of marker proteins for the selected types of endosomes – EEA1 for early endosomes, Rab7 for late endosomes, and LAMP1 for lysosomes. We treated astrocytes with poly(I:C) or poly(I:C)/LyoVec at different concentrations or for various time intervals and performed western blot analysis with the appropriate antibodies.

**FIGURE 1:**
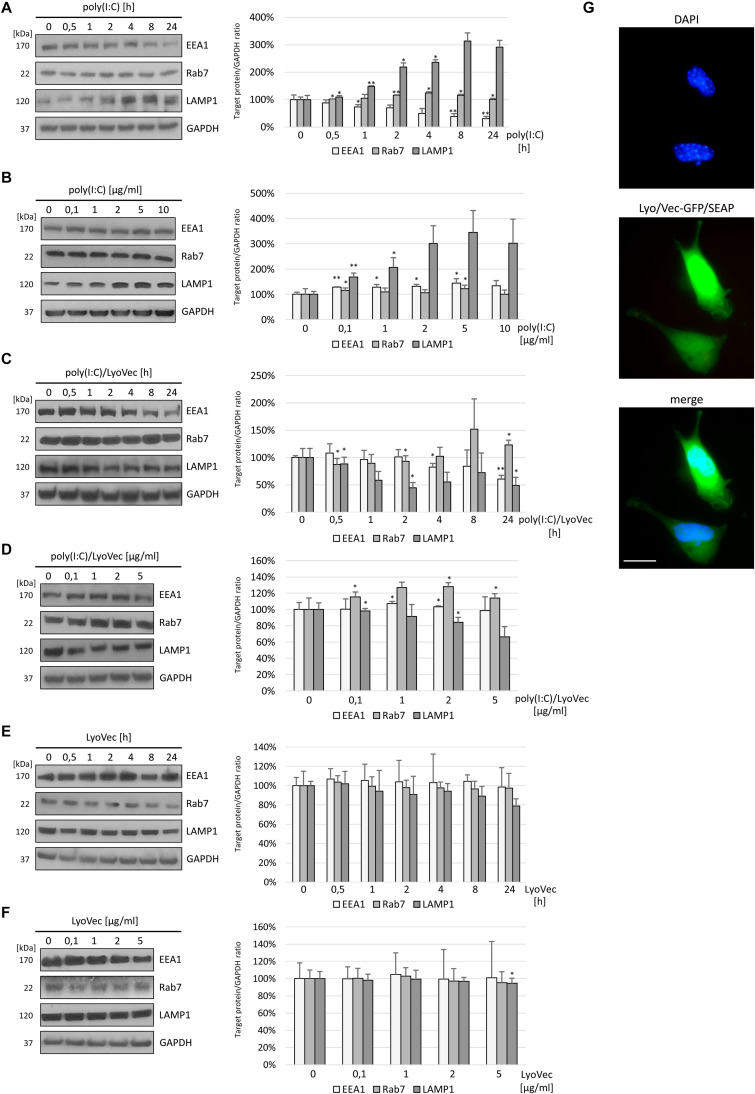
EEA1 decreases and LAMP1 expression increases depending on poly(I:C) stimulation time of C8-D1A murine astrocytes, while increasing TLR3 ligand concentration augments the expression of LAMP1. Representative western blots of EEA1, LAMP1, and Rab7 expression in C8-D1A cells treated with poly(I:C) at concentration 10 μg/ml **(A)**, with poly(I:C)/LyoVec at concentration 1 μg/ml **(C)**, or with LyoVec at concentration 1 μg/ml **(E)**, and lysed at various times following stimulation (0, 30 min, 1, 2, 4, 8, and 24 h). EEA1, LAMP1, and Rab7 expression was also analyzed in cells treated with different concentrations of poly(I:C) (0; 0,1; 1; 2; 5; 10 μg/ml) **(B)**, poly(I:C)/LyoVec (0; 0,1; 1; 2; 5 μg/ml) **(D)**, or LyoVec (0; 0,1; 1; 2; 5 μg/ml) **(F)**, and lysed 24 h following stimulation. GAPDH levels are shown to evaluate equal gel loading. The densitometry analysis of EEA1, LAMP1, and Rab7 was performed in cells treated with 10 μg/ml poly(I:C) for indicated time points **(A)**; indicated poly(I:C) concentrations for 24 h **(B)**; 1 μg/ml poly(I:C)/LyoVec for indicated time points **(C)**; indicated poly(I:C)/LyoVec concentrations for 24 h **(D)**; 1 μg/ml LyoVec for indicated time points **(E)**; or indicated LyoVec concentrations for 24 h **(F)**. Protein expression levels were normalized to GAPDH and presented as relative expression (mean ± SD). The GFP/SEAP reporter was efficiently delivered into C8-D1A cells when LyoVec was used as a transfectant **(G)**. Statistical comparisons were performed between untreated and poly(I:C)- or poly(I:C)/LyoVec- or LyoVec-treated astrocytes (two sample Student’s *t*-test; *P ≤ 0.05; **P ≤ 0.01). Cells transfected with LyoVec alone were used as a control to disengage the contribution of the membrane solubilization as well as interaction of the cationic detergent with cellular compartments. Data were obtained from three independent experiments.

Stimulation of murine astrocytes with the TLR3 agonist resulted in a continuous decrease in EEA1, whereas the expression of Rab7 increased significantly until 4–8 h after poly(I:C) stimulation and then returned to baseline (Figure 1A). Interestingly, after 1 h of poly(I:C) stimulation, LAMP1 increased its expression by 47.8 ± 3.13% (Figure 1A) and generally showed a statistically significant increase of expression. In C8-D1A cells, nuclear translocation of NF-κB was already observed at 8–12 min following stimulation with the TLR3 ligand (Mielcarska et al., 2019), therefore, such a result may indicate a role for LAMP1-expressing endosomes such as late endosomes and lysosomes in mounting TLR3-mediated inflammatory response in astrocytes. As shown in Figure 1B, poly(I:C) treatment resulted in the increase of EEA1 and LAMP1 expression in a concentration-dependent manner, while the increase in Rab7 expression was observed with increasing concentrations of poly(I:C)/LyoVec (Figure 1D). After treatment of C8-D1A cells with poly(I:C)/LyoVec, the expression of EEA1 and LAMP1 decreased, which may be indicative of different cellular events during the stimulation of RIG-I and MDA5 receptors (Figure 1C), and thus may serve as a good control for TLR3 stimulation with poly(I:C). Control experiments with the LyoVec transfection reagent were performed to eliminate the contribution of cell membrane solubilization, which could affect the differences in protein expression observed in experiments with poly(I:C) and poly(I:C)/LyoVec (Figures 1E,F). LyoVec transfection efficiency was confirmed by transfection of C8-D1A cells with LyoVec-GFP/SEAP complex (Figure 1G). Taken together, these results show that C8-D1A astrocytes, besides their responsiveness to the TLR3 ligand, alter the expression pattern of EEA1 and LAMP1, which presumably plays a part in the activation of TLR3-mediated intracellular signaling pathway.

### Astrocytic TLR3 Is Present in EEA1-Labeled Early Endosomes at Least up to 1 h Following Poly(I:C) Stimulation

**FIGURE 2:**
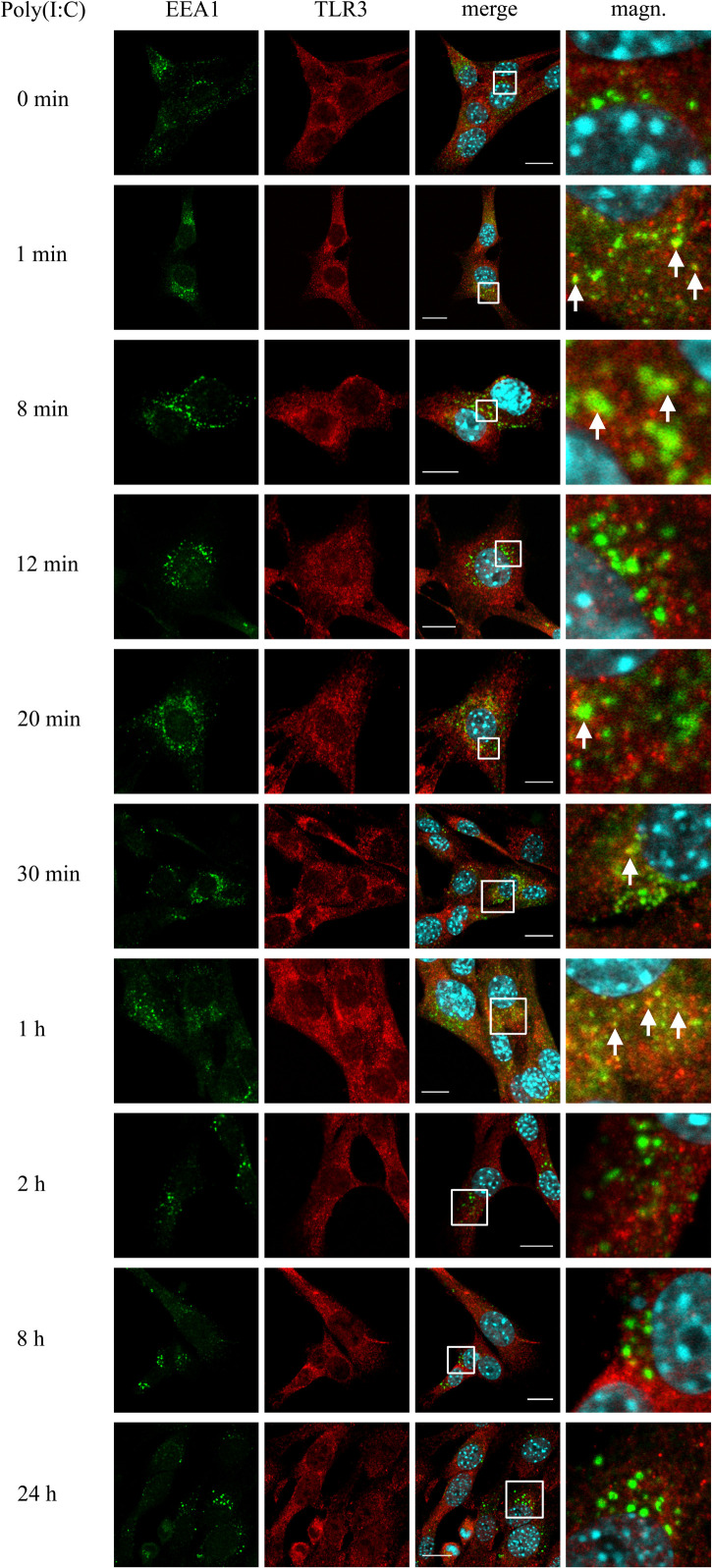
TLR3 is present in EEA1-labeled endosomes at least up to 1 h following poly(I:C) stimulation of murine astrocytes. C8-D1A cells were not treated or treated with poly(I:C) for 1, 8, 12, 20, 30 min, 1, 2, 8, and 24 h. The astrocytes were then fixed and stained with specific antibodies for EEA1 (green) and TLR3 (red). Magn. – magnification of the selection marked with a white frame on the merged image (merge). White arrows indicate the TLR3 and EEA1 co-localization sites. Experiments were performed at least three times and only representative images are shown. Scale bar: 10 μm. Indirect PLA was performed to study the co-localization of EEA1 and TLR3 in not treated cells or in cells following 1 h of poly(I:C) stimulation. Positive PLA (red dots) was observed in cells stimulated with poly(I:C) for 1 h (see Supplementary Figure 2A).

Investigating the role of early endosomes in TLR3 transportation, we examined the localization of TLR3 and EEA1-labeled endosomes in C8-D1A cells unstimulated or stimulated with poly(I:C) (10 μg/ml) for 1, 8, 12, 20, and 30 min, 1, 2, 8, and 24 h. We chose such a time scale, mainly due to the rapidity of the formation of early endosomal vesicles during dsRNA uptake, to observe possible interactions between TLR3 and early endosomes. At the same time, we aimed to obtain evidence on early endosomal events in the astrocytes during the first 24 h after the addition of poly(I:C). In C8-D1A cells, TLR3 association with EEA1-labeled endosomes occurred as early as from the first minute after addition of the TLR3 ligand, suggesting that TLR3 may enter early endosomes already at this time (Figure 2). The most frequent and robust co-localization was observed at 1 and 8 min following cell stimulation, however, at 8 min co-staining was not seen in all cells. At 20, 30 min and 1 h following poly(I:C) treatment, TLR3 only partially co-stained with EEA1. After 1 h, co-localization of TLR3 and EEA1 was no longer detected. Graphs showing the EEA1 and TLR3 co-localization profiles are included in Supplementary Figure 1. Control experiments with TLR3 labeled with FITC, and EEA1 labeled with rhodamine were performed in untreated cells and cells stimulated with poly(I:C) for 1 h. Analogous experiments were conducted using Duolink^®^ *in situ* PLA (Supplementary Figure 2A). In summary, co-localization of the astrocytic TLR3 and EEA1-labeled endosomes occurred during the first hour after the addition of poly(I:C). No co-staining was observed at 2 h and later, indicating that after this time TLR3 may have already been translocated from the early endosomes.

### TLR3 Transits to Late Endosomal Compartment Depending on the Duration of Poly(I:C) Stimulation of Astrocytes

**FIGURE 3:**
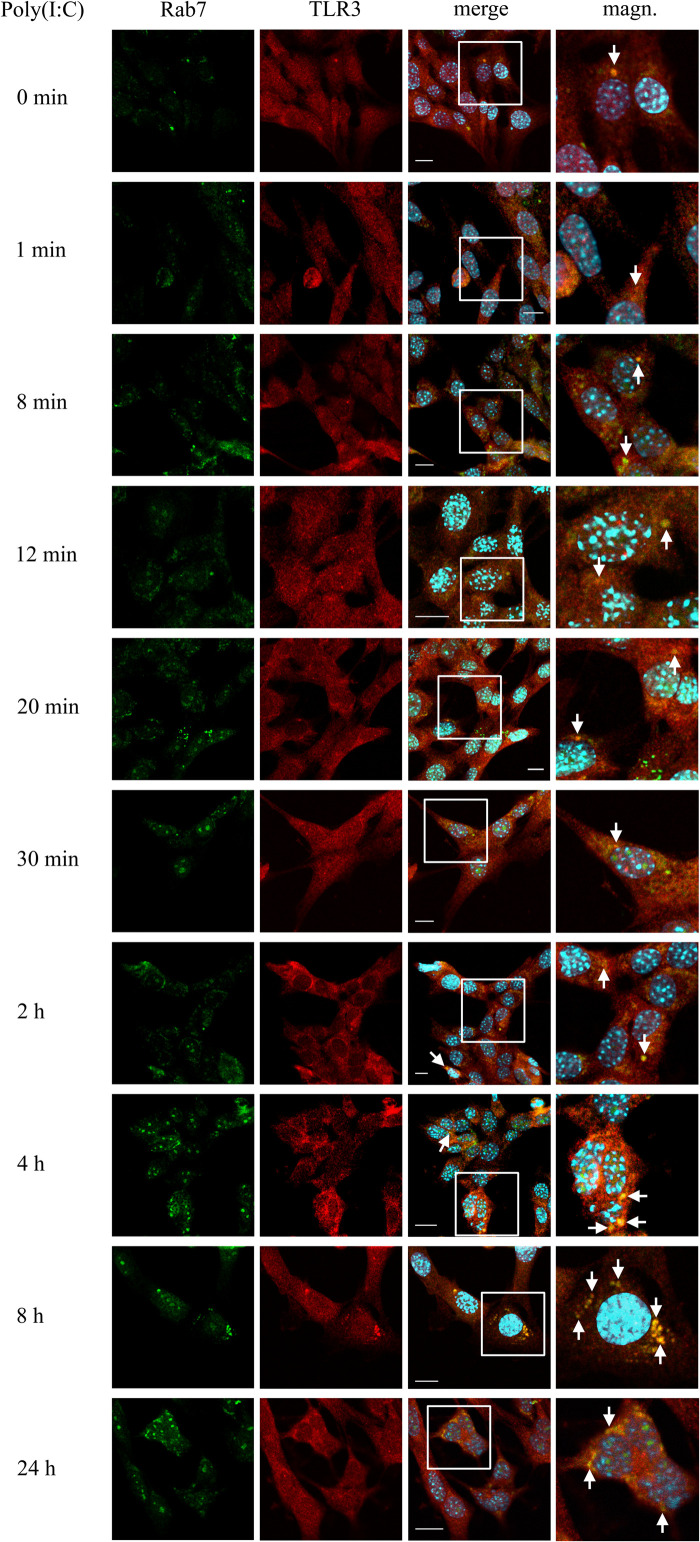
Astrocytic TLR3 is constantly present in late endosomes, however, TLR3 and Rab7 co-localization increases with the time of poly(I:C) stimulation. C8-D1A murine astrocytes were not stimulated or stimulated with poly(I:C) for 1, 8, 12, 20, 30 min, 2, 4, 8, and 24 h. Co-localization of Rab7 (green) and TLR3 (red) occurred in vesicular structures, which can be seen on merged images (merge). All merged pictures have been enlarged in the white frame area where the co-localization of Rab7 and TLR3 can be seen (magn.). White arrows indicate co-localization sites. Experiments were performed at least three times and only representative images are shown. Scale bar: 10 μm. Indirect PLA was performed to investigate the co-localization of Rab7 and TLR3 in untreated astrocytes or in astrocytes treated with poly(I:C) for 24 h. Positive PLA (red dots) was observed in cells stimulated with poly(I:C) for 24 h (see Supplementary Figure 2B).

To investigate the participation of late endosomes in TLR3 transportation, we examined the co-localization of TLR3 and Rab7-labeled endosomes in murine astrocytes not treated or treated with poly(I:C) (10 μg/ml) for 1, 8, 12, 20, 30 min, 2, 8, and 24 h. The time range used here was selected basing on a collection of observations of others, where dsRNA, the TLR3 ligand, was present in Rab7-labeled endosomes in various types of cells at different times after stimulation (Qi et al., 2012; Westphal et al., 2017). In our experiments, the presence of TLR3 in the late endosomes was confirmed at all-time points examined (Figure 3). Noteworthy, until 20 min, TLR3 was present only in individual endosomes in several cells, while most of the late endosomes did not show the presence of TLR3. However, shortly thereafter, the formation and enlargement of endosomes was observed. Separate Rab7-labeled endosomes appeared in astrocytes at 30 min, while at 8 and 24 h we observed prominent, abundant co-localization of TLR3 and late endosomes (Figure 3). Interestingly, from the first minute following poly(I:C) stimulation, a significant part of Rab7 was localized in what we think is the nucleoli, sites of the ribosome biogenesis. However, this requires experimental confirmation. Control experiments with TLR3 labeled with FITC, and Rab7 labeled with rhodamine were performed in untreated astrocytes and astrocytes treated with poly(I:C) for 24 h. Similar experiments were conducted using Duolink^®^ *in situ* PLA (Supplementary Figure 2B). Our data suggest that as the stimulation time increases, TLR3 is more abundant in late endosomes, with the highest presence at 8 and 24 h after the addition of poly(I:C).

### TLR3 Largely Co-localizes With LAMP1-Labeled Lysosomes Following Poly(I:C) Stimulation of Murine Astrocytes

**FIGURE 4:**
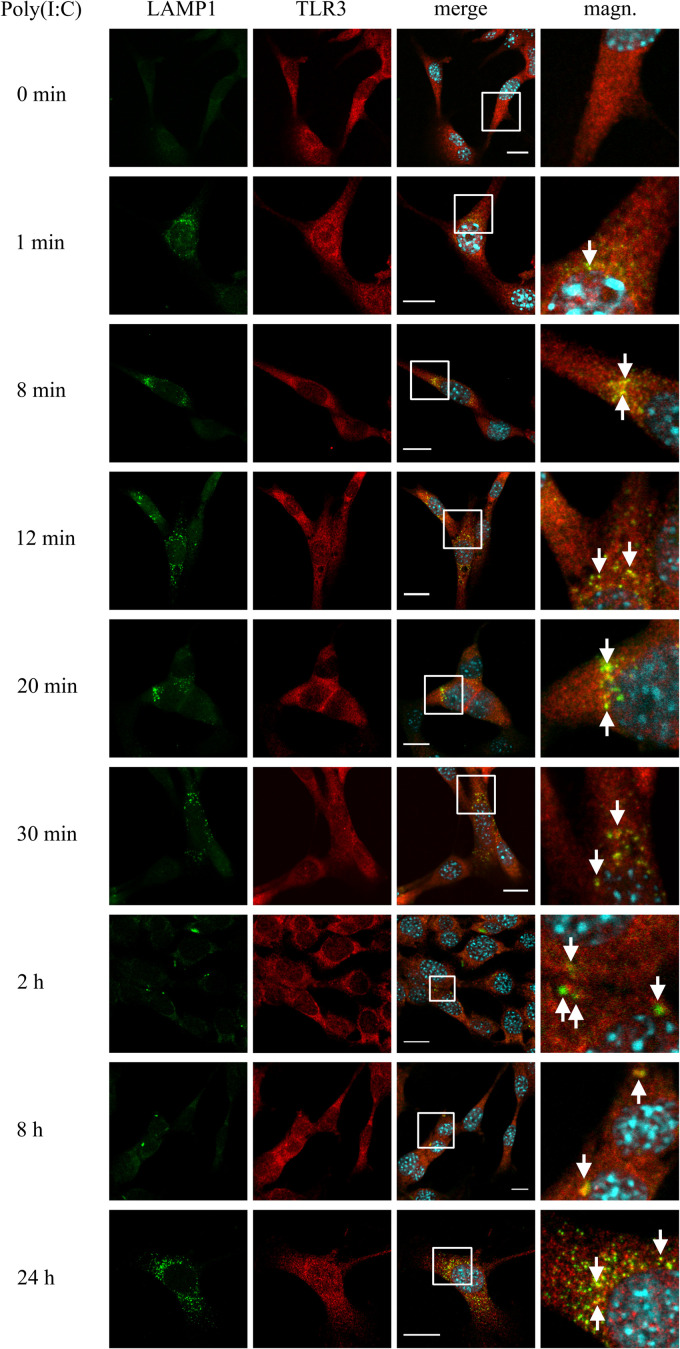
TLR3 is abundantly translocated to LAMP1-labeled endosomes after poly(I: C) stimulation of C8-D1A cells. Mouse astrocytes were not treated or treated with poly(I:C) for 1, 8, 12, 20, 30 min, 2, 8, and 24 h. The cells were then fixed and labeled with anti-LAMP1 (green) and anti-TLR3 (red) antibodies. All merged images (merge) have been enlarged in the area where the co-localization of LAMP1 and TLR3 can be seen (magn.) and white arrows indicate co-localization sites. Experiments were performed at least three times and the representative images are shown. Scale bar: 10 μm. To explore the co-localization of LAMP1 and TLR3 in untreated cells or in cells following 24 h of poly(I:C) stimulation, indirect PLA was performed. Positive PLA (red dots) was observed in cells stimulated with poly(I:C) for 24 h (see Supplementary Figure 2C).

Studies on distinct cell lines demonstrate that TLR3 senses dsRNA in endosomes, in which the pH varies from 6.0 (early endosomes) to 5.5 or below (late endosomes and lysosomes). The affinity of TLR3 to its ligand increases with the environment acidity (Leonard et al., 2008), however, the half-life of the receptor in the endolysosomal compartment remains variable. To examine the potential time of TLR3 residence in the lysosomal compartment, we examined the co-localization of TLR3 and LAMP1-labeled endosomes in C8-D1A cells not treated or treated with poly(I:C) (10 μg/ml) for 1, 8, 12, 20, 30 min, 2, 8, and 24 h. In resting astrocytes, TLR3 did not co-stain with lysosomes, however, the presence of the receptor was observed in LAMP1-marked endosomes in the first minute following treatment of cells with poly(I:C), and in all other times tested (Figure 4). Thus, astrocytic TLR3 is not constitutively present in LAMP1(+) endosomes, but the localization of the receptor changes in response to its ligand. In the first minute after poly(I:C) stimulation, TLR3 appears to begin the translocation into LAMP1-labeled endosomes, because between 8 and 30 min co-localization of TLR3 and LAMP1 increased. Furthermore, the studied vesicles were initially spread throughout the cell, compared to those at 2 and 8 h post-stimulation, where TLR3 is present in single endosomes, however, only in some astrocytes. Finally, 24 h after poly(I:C) addition, we detected numerous lysosomes in all astrocytes and abundant co-localization with TLR3. Graphs showing the LAMP1 and TLR3 co-localization profiles are included in Supplementary Figure 3. Control experiments with TLR3 labeled with FITC, and LAMP1 labeled with rhodamine were conducted in untreated cells and cells treated with poly(I:C) for 24 h. Corresponding experiments were performed using Duolink^®^ *in situ* PLA (Supplementary Figure 2C). Therefore, the presence of TLR3 in LAMP1-marked endosomes is ligand-dependent, and TLR3 can be found in the lysosomal compartment as early as the first minute following cell treatment. There is a possibility that these endosomes serve as TLR3 signaling compartment, however, they may also participate in TLR3 turnover and function as a safeguard to balance the signaling level.

### TLR3 Interacts With dsRNA From the First Minute Following Stimulation of Astrocytes With Poly(I:C)

**FIGURE 5:**
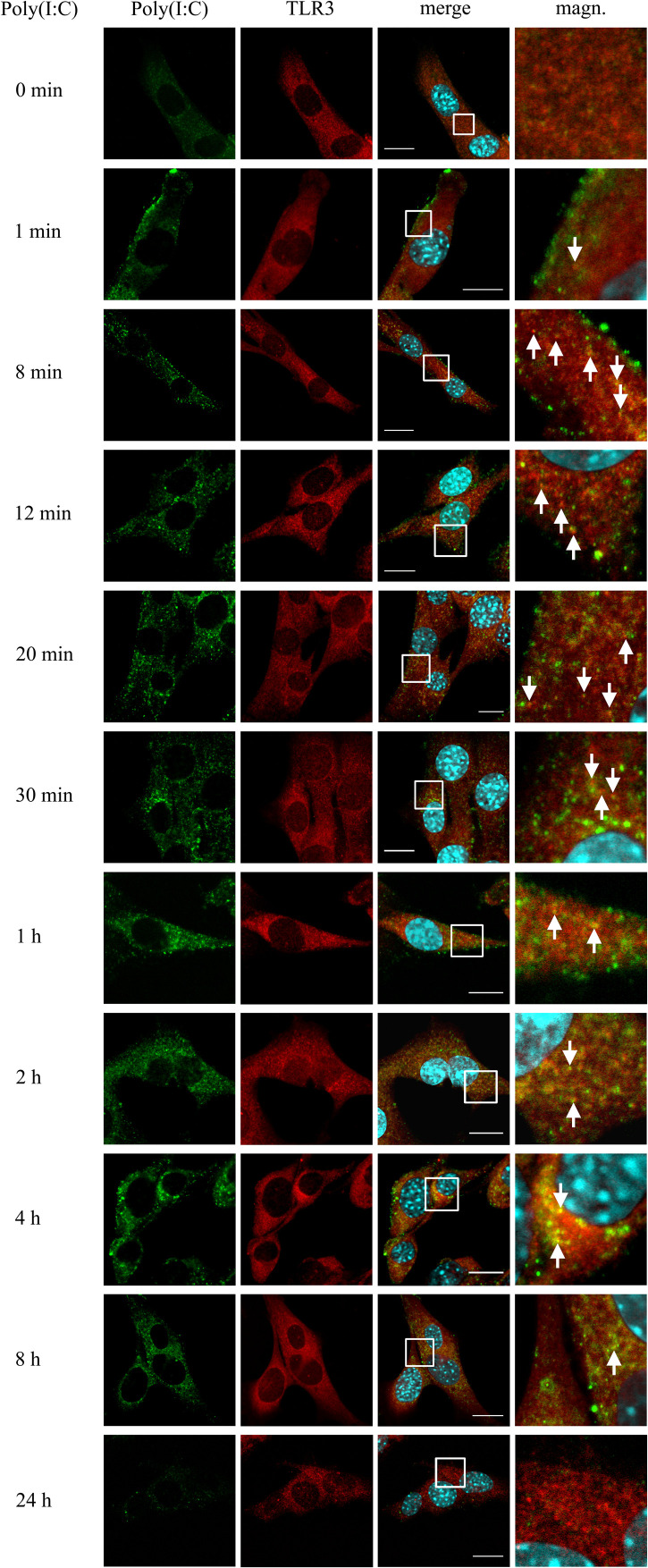
TLR3 interacts with poly(I:C) from the first minute following stimulation of C8-D1A cells with the TLR3 ligand. C8-D1A cells were not stimulated or stimulated with poly(I:C) for 1, 8, 12, 20, 30 min, 1, 2, 4, 8, and 24 h. The astrocytes were then fixed and labeled with anti-dsRNA (green) and anti-TLR3 (red) antibodies. All merged images (merge) have been enlarged in the area where the co-localization of poly(I:C) and TLR3 can be seen (magn.). White arrows indicate the TLR3 and poly(I:C) co-localization sites. Experiments were performed at least three times and the representative images are shown. Scale bar: 10 μm.

TLR3 senses dsRNA and initiates immune response through the emergence of the signaling unit consisting of one dsRNA and two TLR3 molecules (Luo et al., 2012). To illustrate the possible TLR3 activation process in the C8-D1A cell line, we studied the interaction of TLR3 with dsRNA in cells stimulated with poly(I:C) (10 μg/ml) for 1, 8, 12, 20, 30 min, 1, 2, 4, 8, and 24 h, while untreated cells served as control. From the first minute following the addition of the TLR3 ligand, poly(I:C) visibly adhered to the cell membranes, which was not observed later than 1 h after stimulation of astrocytes (Figure 5). TLR3 and dsRNA co-localization was also noticeable from the first minute following the addition of poly(I:C), however, the highest abundance of the TLR3 and poly(I:C) interaction was observed between 8 min and 8 h after cell stimulation. Interestingly, we did not observe TLR3 and dsRNA co-localization 24 h after the addition of poly(I:C), however, at this time, the receptor largely interacted with protein markers of late endosomes and lysosomes (Figures 3, 4). Such TLR3 interaction with these types of endosomes may preferentially indicate targeting of the receptor for the degradation. Taken together, TLR3 interaction with the ligand was observed in the interior of astrocytes as early as from the first minute after the addition of poly(I:C) and lasted up to 8 h following cell stimulation.

### TLR3 of Murine Astrocytes Does Not Shuttle to Endosomes Following Treatment With CU CPT 4a, and Subsequent Stimulation With Poly(I:C)

**FIGURE 6:**
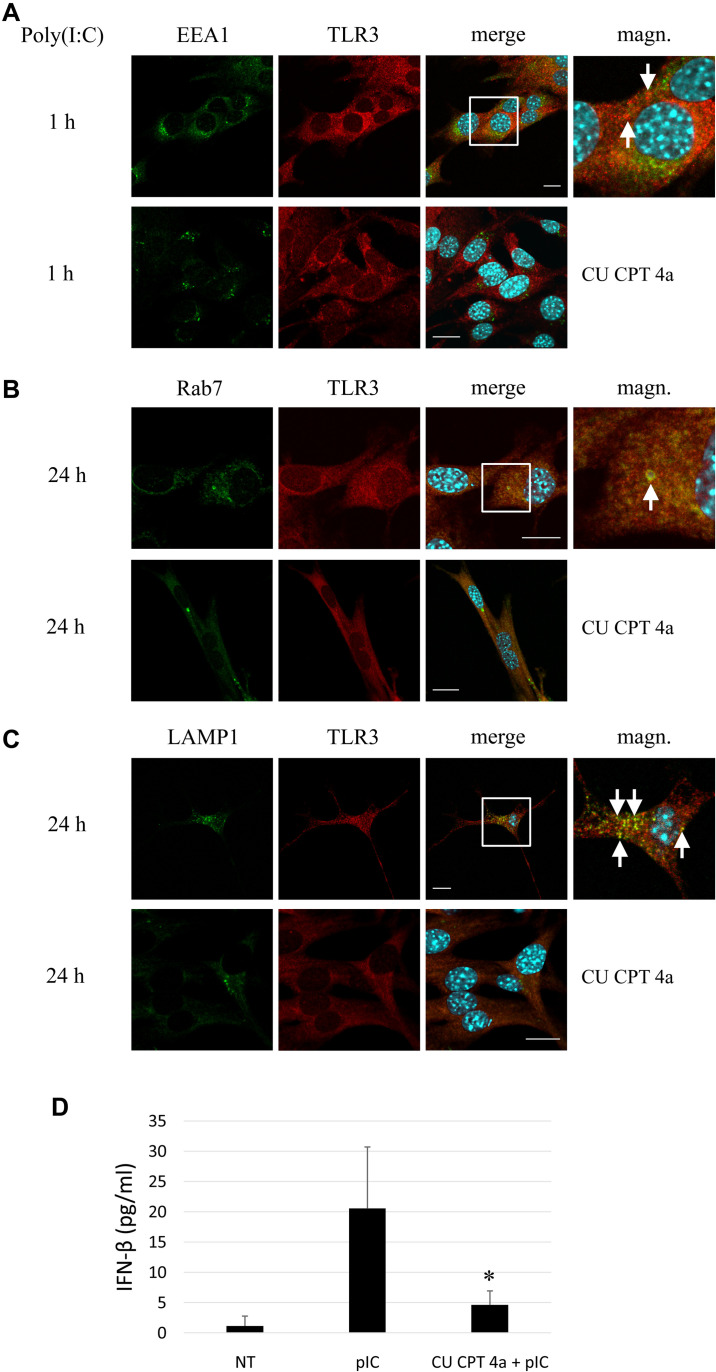
Astrocytic TLR3 is not present in early endosomes, late endosomes or lysosomes following pretreatment with the TLR3 inhibitor, CU CPT 4a, and subsequent poly(I:C) stimulation. C8-D1A murine astrocytes were stimulated with poly(I:C) for 1 or 24 h. Co-localization of EEA1 **(A)**, Rab7 **(B)** or LAMP1 **(C)** (green) and TLR3 (red) occurred in vesicular structures, however, no interaction of the studied proteins was observed following treatment of cells with CU CPT 4a (20 μM) for 1 h before the addition of poly(I:C). Merged images (merge) have been enlarged in the area where the co-localization of EEA1 and TLR3 **(A)**, Rab7 and TLR3 **(B)**, and LAMP1 and TLR3 **(C)** can be seen (magn.) and white arrows indicate co-localization sites. **(D)** ELISA assay result demonstrates that CU CPT 4a significantly inhibits TLR3 and subsequently IFN-β secretion following stimulation with 10 μg/ml poly(I:C) in C8-D1A cells. Statistical comparisons were performed between untreated and poly(I:C)- or CU CPT 4a and poly(I:C)-treated astrocytes (two sample Student’s *t*-test; *P ≤ 0.05; **P ≤ 0.01). Experiments were performed at least three times and only representative images are shown. Scale bar: 10 μm.

To produce evidence that poly(I:C) selectively activates TLR3 signaling pathway in murine astrocytes, we used CU CPT 4a, repressor of the downstream signaling mediated by the TLR3/dsRNA complex. C8-D1A cells were treated with CU CPT 4a for 1 h and then stimulated with poly(I:C). Subsequently, the interaction of TLR3 with EEA1, Rab7 and LAMP1 was studied. Co-localization of TLR3 labeled with rhodamine, and EEA1 labeled with FITC was examined in cells stimulated with poly(I:C) for 1 h (Figure 6A); while interaction of TLR3 labeled with rhodamine and Rab7 or LAMP1 labeled with FITC was studied in astrocytes stimulated with poly(I:C) for 24 h (Figures 6B,C). With the absence of CU CPT 4a pretreatment, poly(I:C)-induced TLR3 co-localization with the endosomal proteins was preserved, as shown in previous experiments (Figures 2–4). The addition of CU CPT 4a resulted in the absence of the TLR3 co-localization with all endosome marker proteins. Furthermore, our results demonstrate that CU CPT 4a is a potent inhibitor of the IFN-β secretion by C8-D1A cells (Figure 6D).

### TLR3 Is Expressed on the Cell Surface of Astrocytes, Either Stimulated With Poly(I:C) or Not Stimulated

**FIGURE 7:**
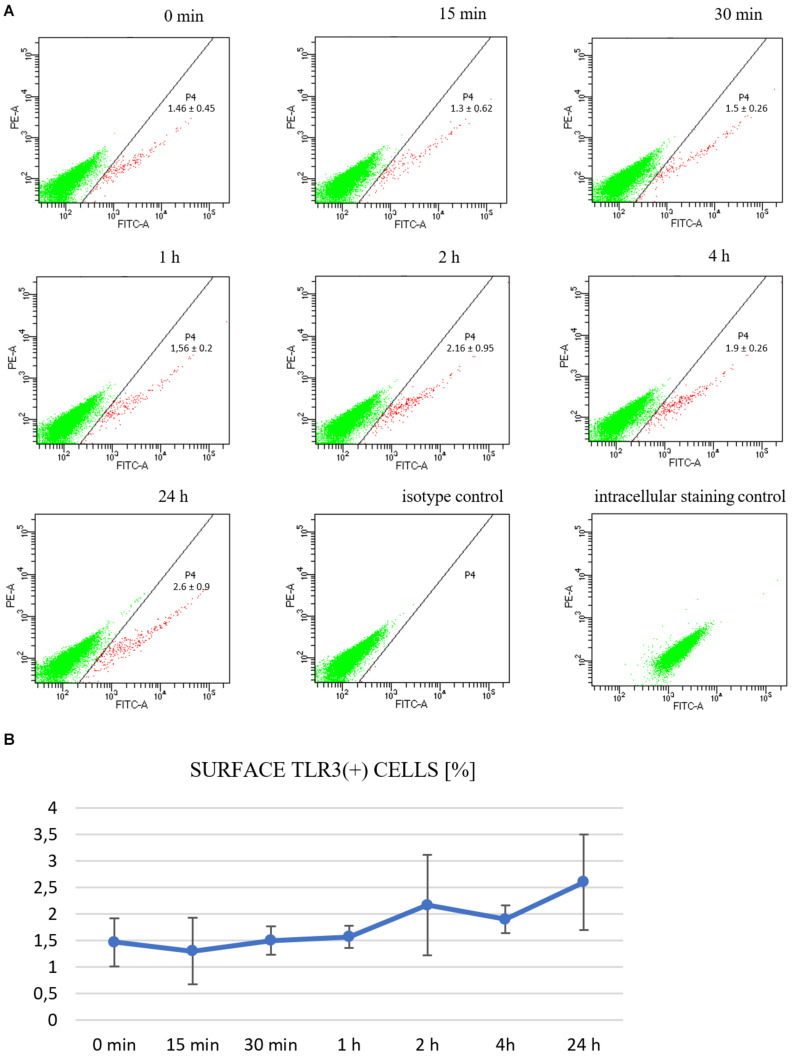
TLR3 is constitutively expressed on the surface of C8-D1A murine astrocytes and is enhanced following stimulation with the TLR3 agonist. Representative cytograms show surface TLR3 protein expression on the cells: not treated with poly(I:C); or treated for 15, 30 min, 1, 2, 4, 24 h; isotype control; intracellular staining control **(A)**. The isotype control serves as the negative control and functions to validate the result that TLR3 present on the surface of astrocytes is not an artifact. The intracellular staining control serves as the positive control to validate the specific anti-TLR3 antibodies used in the experiment by staining the intracellular TLR3, following permeabilization of the cells. P4 – astrocyte population where TLR3 occurs on the surface. Numbers in the dot plots represent the mean percentage ± SD of surface TLR3(+) cells in the three independent experiments. The cytograms show green fluorescence on the *x*-axis and red fluorescence on the *y*-axis. **(B)** Graph showing the occurrence of TLR3 on the cell surface at various times following poly(I:C) treatment (% of all cells). Statistical comparisons were performed between untreated and poly(I:C)-treated astrocytes (two sample Student’s *t*-test; *P ≤ 0.05; **P ≤ 0.01). Experiments were performed three times, and each dot plot represents mean ± SD. The graph shows the time of astrocytic stimulation with poly(I:C) on the *x*-axis, and the percentage of cells where TLR3 occurred on the surface on the *y*-axis.

Although the endosomal acidic environment is crucial for the functioning of endosome-populating TLRs, in particular cells, TLR3 may exist as a functional receptor on the cell surface (Hewson et al., 2005; Groskreutz et al., 2006; Murakami et al., 2014). To investigate whether TLR3 is expressed on the plasma membrane of murine astrocytes, we immunostained non-permeabilized cells with antibodies directed against the extracellular domain of the TLR3 protein and performed cytometric analysis. Cells were previously unstimulated or stimulated with poly(I:C) for 15, 30 min, 1, 2, 4, or 24 h. TLR3 staining was detected on the surface of all tested live gated cells (Figure 7A). The frequency of surface TLR3(+) cells varied from 0.8 to 3.5% of live gated C8-D1A cell preparations, compared to the isotype control (0 to 0.1%). Furthermore, the proportion of surface TLR3(+) cells correlated with the time of stimulation with poly(I:C), however, obtained results were not statistically significant (Figure 7B).

## Discussion

TLR3 is involved in the sensing of dsRNA, the replication intermediate of many DNA and RNA viruses (Table 2), and regulates immunity to most of the clinically important viral infections in humans (Perales-Linares and Navas-Martin, 2013). Although molecular pathways that lead to TLR3 activation have been largely explained, the knowledge regarding TLR3 intracellular transportation, accession to the ligand recognition site, and degradation is still lacking. The presence of the receptor has been confirmed in various types of endosomes and studies referenced in this discussion demonstrate that cellular localization of TLR3 depends considerably on the studied cell type, which has been highlighted in Marek and Kagan (2011) review. In our experiments on murine astrocytes, we confirmed the presence of TLR3 in all types of endosomes studied, although the frequency and intensity of TLR3 co-localization with EEA1, Rab7, and LAMP-1 varied depending on the duration of astrocyte stimulation with synthetic dsRNA. During the first ten minutes following poly(I:C) stimulation, astrocytic TLR3 was abundant in EEA1-labeled endosomes and appeared readily in LAMP1-labeled vesicles (Figures 2, 4 and Supplementary Figures 2A,C), while the peak of TLR3 presence in late Rab7-labeled endosomes occurred between 8 and 24 h after poly(I:C) addition (Figure 3). Finally, 24 h after the addition of poly(I:C), TLR3 co-localized with lysosomes in all astrocytes. TLR3 is an evolutionarily conserved molecule, constitutes a separate family among six major families of vertebrate TLRs (Roach et al., 2005), and can occur in the same endosome types in both mammals and invertebrates (Zhang et al., 2013). Our opinion is that discrepancies in populating various endosomes by TLR3 at different time intervals after stimulation of astrocytes reflect the stages of TLR3 transportation and the diverse functions of endosomes.

**TABLE 2:** Viruses that induce TLR3 signaling leading to generation of a protective response.

Virus	Host	Genetic material	Source
HSV-1	Human	dsDNA	Zhang et al. (2007); Guo et al. (2011), Lafaille et al. (2012)
HSV-1	Mouse	dsDNA	Reinert et al. (2012)
IAV	Human	ssRNA segments	Hidaka et al. (2006)
HIV-1	Human	ssRNA	Fujimoto et al. (2004); Rivieccio et al. (2006), Swaminathan et al. (2012)
HCV	Human	ssRNA	Li et al. (2012)
Human PV	Mouse	ssRNA	Abe et al. (2012)
HSV-2	Mouse	dsDNA	Reinert et al. (2012)
West Nile Virus	Mouse	ssRNA	Daffis et al. (2008)
CVB3	Mouse	ssRNA	Abston et al. (2012)
CVB4	Mouse	ssRNA	Richer et al. (2009)
EMCV	Mouse	ssRNA	Hardarson et al. (2007)
MCMV	Mouse	dsDNA	Hoebe et al. (2003); Tabeta et al. (2004)
TMEV	Mouse	ssRNA	Jin et al. (2011)

*For DNA viruses the response is induced by the RNA intermediates that are generated during replication.*

By sorting, processing, recycling, activating, silencing, and degrading distinct membrane proteins including receptors, endosomes are responsible for the regulation of numerous signaling pathways in the cell (Huotari and Helenius, 2011). During endosome biogenesis, various sets of proteins are sequentially acquired. An important element in the course of endosome maturation process is the loss of early endosomal marker EEA1, and the recruitment of Rab7, a marker of late endosomes (Rink et al., 2005). Subsequently, progressive endosomal acidification occurs through fusion with lysosomal compartments and LAMP1 is one of the most abundant protein components in lysosome membranes (Terasawa et al., 2016). Because TLR3 populates various endosomes at different time intervals after the addition of poly(I:C), we postulated that one of the important challenges during the receptor localization study is to assess the appropriate time scale following the receptor activation.

During the formation of early endosomes, the endocytosed material, e.g., extracellular dsRNA (Tatematsu et al., 2018) is packed within approximately 8–15 min into early endosomes, which merge in the perinuclear region and form late endosomes/multivesicular bodies (MVB) (Huotari and Helenius, 2011). In our studies, TLR3 localized to early endosomes during the first hour after stimulation of astrocytes with the TLR3 ligand. Co-localization of TLR3 and EEA1 in astrocytes shows consistency with results in the other report on cells other than astrocytes. In human myeloid dendritic cells (mDCs) EEA1-labeled early endosomes co-localized with fluorescently labeled dsRNA 15 and 30 min, however, not as early as 5 min following cell stimulation (Johnsen et al., 2006). Accordingly, early endosomes may perform a transport function for dsRNA as well as deliver TLR3 to other populations of endosomes for proteolytic processing and/or activation. Other studies corroborate co-localization of early endosomes with TLR3 following stimulation of HeLa cells with IFN-β (Tatematsu et al., 2015), or following the receptor stimulation in human monocyte-derived immature DCs (MoDCs) (Watanabe et al., 2011) and 293T cells (Yang et al., 2016). Nevertheless, in NCI-H292 cells not treated or treated with poly(I:C) for 30, 60, and 120 min, TLR3 did not co-localize with EEA1 (Toscano et al., 2013), and in bone marrow-derived macrophages (BMDMs) TLR3 co-localized with early endosomes 2 and 4 h following poly(I:C) stimulation (Tsai et al., 2015). Therefore, the role of early endosomes in mediating the transportation of membrane proteins such as TLR3 and TLR3 ligands is worth studying.

Late endosomes increase in size by incorporating early endosomes and lysosomal hydrolases. These hybrid organelles mediate the trafficking of lysosomal components and liaise protein transportation from the *trans*-Golgi network (TGN) to lysosomes (Huotari and Helenius, 2011). In our work, we observed the coexistence of TLR3 and Rab7 in resting astrocytes as well as following stimulation with the TLR3 agonist, with the most abundant co-localization at 8 and 24 h after poly(I:C) addition. Westphal et al. (2017) demonstrated activation-induced Rab7(+) endosomal compartment as a major site of TRIF-mediated signaling events, which include the TLR3 pathway. Tsai et al. (2015) also considered Rab7-labeled late endosomes as crucial for the TLR3 activation, since blockade of these endosomes resulted in the loss of IFN-β expression and production. Interestingly, the inhibition of TLR3 proteolytic processing increased TLR3 co-localization with endosomes marked with Rab7 and LAMP1 (Qi et al., 2012). These results indicate that late endosomes can play an important role in TLR3 activation, as they contain components from early endosomes and provide an environment adequate for the activity of cathepsins, enzymes crucial for TLR3 signaling (Garcia-Cattaneo et al., 2012). However, late endosomes may also be involved in receptor degradation. The abundant co-localization of TLR3 with Rab7 at 8 and 24 h after receptor stimulation may indicate the involvement of endosomes in this process, as signal cascade and translocation of relevant transcription factors to the cell nucleus take place within 15 min after stimulating astrocytes with poly(I:C) (Mielcarska et al., 2019) and the degradation could occur at the same time or later following TLR3 stimulation. Negative regulation of TLR3 may account for the process parallel to signaling, similar to cleavage events following TLR9 activation (Hasan et al., 2016; Sinha et al., 2016). In our opinion, TLR3 signaling accompanied by targeted loss of the receptor would aim at maintaining TLR3 homeostasis.

The translocation of TLR3 to lysosomes occurred from the first minute after the addition of poly(I:C) to astrocytes. Such a result is consistent with that in human mDCs, where TLR3 trafficked into tubular lysosomes labeled with LAMP1 30 min after cell stimulation, but no co-localization was observed before this time (Johnsen et al., 2006). Other studies also corroborate TLR3 presence in LAMP1-labeled endosomes 30 min following stimulation of 293T cells (Yang et al., 2016). The prompt emergence of TLR3 in lysosomes following treatment of cells with poly(I:C) possibly occurs due to the receptor activation. We speculate that LAMP1-labeled endosomes constitute a TLR3 ligand recognition site, however, confirmatory studies are essential to incorporate this hypothesis. Interestingly, Toscano et al. (2013) observed that TLR3 abundantly co-localized with LAMP1, regardless of the epithelial cell stimulation, and the receptor levels remained uniform in LAMP1-marked endosomes. Our studies show that the highest abundance of TLR3 in LAMP1 endosomes was achieved after 24 h of stimulation (Figure 4 and Supplementary Figure 2C) while no interaction of TLR3 and poly(I:C) was confirmed (Figure 5). At this time, similar to TLR9, we might observe a TLR3 fragment that remains in lysosomes and could perform an autoregulatory function (Lee et al., 2014). This is congruent with another study regarding TLR3 intracellular localization, which showed that TLR3 did not co-localize with the lysosome marker in resting DCs, however, 24 h after poly(I:C) stimulation, the receptor extensively shuttled to LAMP1(+) endosomes (Jelinek et al., 2011). Such receptor localization 24 h after ligand addition may also indicate its degradation. Lysosomal degradation is vital in cell regulation and counterbalances the extension of the signaling (Huotari and Helenius, 2011), however, more investigation is required to elucidate the fate of TLR3 as lysosomal cargo including inactivation of the receptor.

Studies on the interaction of poly(I:C) and TLR3 allowed us to conclude that an important research quandary remaining to be addressed when identifying the TLR3-containing compartment is the TLR3 ligand adsorption time and fate in cells. In C8-D1A astrocytes, fluorescein-labeled poly(I:C) localized intracellularly 16 h after addition to the cell medium (data not shown), however, conjugation of dsRNA with the fluorescent label may result in different absorption kinetics in the cells than unlabeled dsRNA. In BMDMs TLR3 co-stained with poly(I:C) at 8 h, but not 2, 10, or 24 h following poly(I:C) addition (Tsai et al., 2015). Conversely, in C8-D1A cells, the interaction of TLR3 and dsRNA was observed right after the addition of the TLR3 ligand up to 8 h following poly(I:C) stimulation (Figure 5). Presumably, poly(I:C) endocytosis occurs in astrocytes within seconds or minutes, resulting in rapid ligand recognition by TLR3 and triggering an antiviral response. On the other hand, the lack of TLR3 and dsRNA interaction 24 h after ligand addition, with the abundant presence of the receptor in lysosomes, may be a sign of an intracellular process related to the negative regulation of the receptor level. We share the point of view that during viral infection, naked viral dsRNA reaches extracellular milieu following lysis of the infected cells (Matsumoto and Seya, 2008; Pohar et al., 2013; Mielcarska et al., 2018), and TLR3 requires the internalization of RNA for sensing through an endocytic pathway (Tatematsu et al., 2018). Several membrane proteins such as the receptor for advanced glycation end-products (RAGE) and CD14 may be involved in the uptake of dsRNA in various cell types and play a role in TLR3-mediated immune signaling (Lee et al., 2006; Bertheloot et al., 2016). Considerable information available through insect research suggests that clathrin-mediated endocytosis (CME), an evolutionarily primeval system, serves as the mechanism responsible for dsRNA uptake, and transports it to early endosomes and subsequently late endosomes (Xiao et al., 2015; Cappelle et al., 2016; Pinheiro et al., 2018). During CME in mammalian cells, plasma membrane scission leads to vesicle formation within ∼10 s (Lacy et al., 2018) and more than half of the particles pass through the cell membrane in the first half of an hour (Parlea et al., 2016). In Sf9 insect cells fluorescein-labeled dsRNA was detected on the plasma membrane 1 min following cell stimulation. Consecutively, uptake of labeled dsRNA was detected within 5 min after the addition of dsRNA to the medium, which eventually accumulated in acidic bodies 10 and 20 min after cell stimulation. Interestingly, dsRNA occurred mainly in early and late endosomes, but not in lysosomes (Yoon et al., 2017), therefore, these vesicles were regarded as dsRNA intracellular carriers.

In our work, for the first time we observed the presence of TLR3 on the surface of murine astrocytes and following poly(I:C) stimulation of the cells the expression of surface TLR3(+) increased (Figure 7). Although only 2.6% of cells reveal TLR3 on the surface following 24 h stimulation with poly(I:C), we speculate that even minor quantities of the surface receptor in individual astrocytes could provide a substantial immune response. However, because we used the antibody raised against TLR3 ectodomain, there is a possibility that another TLR was also visualized, due to the notable sequence homology between the TLR extracellular domains (Marek and Kagan, 2011). Moreover, studies with a virus that expresses a dsRNA intermediate have yet to be performed to compare poly(I:C) effect with the virus. HSV-1 infects primary murine glial cells, among which astrocytes are the most readily infected (Aravalli et al., 2006). Interestingly, in cultures of glial cells, astrocytes may increase TLR3 expression following interaction with activated microglia; therefore, it is worth conducting an additional study in glial cell co-cultures. Although TLR3 is primarily found in the intracellular site, the receptor also occurs on the surface of mast cells (Agier et al., 2016), macrophages (Murakami et al., 2014), primary human astrocytes (Bsibsi et al., 2002), and in the plasma membrane of epithelial cells where it serves as a defensive front line in the bronchial or gastrointestinal tract (Hewson et al., 2005; Bugge et al., 2017). Moreover, rhinovirus (RV) and respiratory syncytial virus (RSV) replication contributed to the increase of TLR3 cell membrane expression (Hewson et al., 2005; Groskreutz et al., 2006). These findings indicate that dsRNA present in the external environment of the cell may interact with TLR3 localized at the plasma membrane and an increase in TLR3 membrane localization may serve to raise the cell sensitivity to viral exposure.

The effort has been made to highlight the possible role of TLR3 and viral encephalitis in the pathogenesis of amyloidosis. Furthermore, the potential of astrocytes to maintain the physiological condition of neurons is particularly important, as these cells may have critical relevance on the prevention of amyloid-β (Aβ) formation and plaque production in the brain. Astrocytes have been shown to secrete Apolipoprotein E (ApoE), which is involved in maintaining normal homeostasis and influences Aβ clearance in the brain (Gee and Keller, 2005; Verghese et al., 2013). However, astrocytes may also contribute to the Aβ burden in the brain by secreting significant quantities of the amyloid, e.g., during conditions such as neuroinflammation (Frost and Li, 2017). Because astrocytes comprise about 90% of the mass of the human brain, defects in their functioning may significantly contribute to brain pathology and particularly to neurodegenerative diseases such as Alzheimer’s disease (AD). HSV-1 is considered as a causative agent in the pathogenesis of sporadic AD (Harris and Harris, 2018), and recent studies suggest that astrocytes secrete Aβ as an anti-microbial protein (AMP) in response to HSV infection (Bourgade et al., 2015). Extensive research data obtained from AD brains and HSV-infected cell cultures demonstrate that latent or chronic brain infections by HSV-1 may induce AD pathophysiology and pathology (Itzhaki, 2017, 2018; Devanand, 2018). Because TLR3 plays a pivotal role in the innate immune response to HSV-1 infection of the CNS, deciphering TLR3 biology and understanding how the receptor journeys over the endosomal pathway in astrocytes are critical to the design of interventional therapeutic options.

In conclusion, we confirm TLR3 intracellular localization in early, late endosomes and particularly in lysosomes in the C8-D1A murine astrocyte cell line. TLR3 was rapidly transported to EEA1-labeled early endosomes and LAMP1-labeled lysosomes after synthetic dsRNA addition. As the stimulation increased, TLR3 largely co-localized with Rab7-labeled late endosomes, with the highest abundance at 8 and 24 h following poly(I:C) stimulation. Further, 24 h after the addition of the TLR3 ligand, the receptor was abundant in lysosomes in all astrocytes. TLR3 interacted with the synthetic ligand from 1 min to 8 h following stimulation of cells. Finally, we demonstrate a constitutive cell surface expression of TLR3 on astrocytes, which is enhanced by poly(I:C) stimulation. We are aware that astrocytes cultured *in vitro* partially acquire a reactive phenotype, therefore, comparable experiments performed on the cells isolated *ex vivo* would yield valuable results. TLR3 traverses the endosomal pathway to anchor in the ligand recognition sites and eventually undergoes degradation. On one hand, intracellular sequestration of TLR3 is requisite to the prevention of accidental recognition of self-nucleic acid, however, TLR3 present in the cell membrane may emerge as a promising target for immune response modulation. Identification of the temporal and spatial model of the TLR3 intracellular residence may also provide a basis for inhibiting or enhancing the TLR3 response.

## Data Availability Statement

The original contributions presented in the study are included in the article/Supplementary Material, further inquiries can be directed to the corresponding authors.

## Author Contributions

MM and FT conceptualized and designed the study. MM, MB-N, KG-Z, and ZW performed western blot analysis. MM, PK, JC, MC, and MMG conducted fluorescent confocal microscopy experiments. KG-Z, LS-D, and MM performed flow cytometry analysis. MM and KG-Z performed ELISA and Duolink^®^ *in situ* PLA. MM, LS-D, FT, and MG analyzed and interpreted the data. LS-D prepared figures. MM and FT wrote the manuscript. All authors contributed to manuscript revision, read, and approved the submitted version.

## Conflict of Interest

The authors declare that the research was conducted in the absence of any commercial or financial relationships that could be construed as a potential conflict of interest.

## Abbreviations

Aβ: amyloid-β
AD: Alzheimer’s disease
ApoE: apoliprotein E
BBB: blood–brain barrier
BMDMs: bone marrow-derived macrophages
BSA: bovine serum albumin
CME: clathrin-mediated endocytosis
CNS: central nervous system
COPII: coat protein II
COVID-19: coronavirus disease 2019
CXCL: C-X-C motif chemokine ligand
dsRNA: double-stranded RNA
EEA1: early endosome antigen 1
ER: endoplasmic reticulum
ESCRT-0: endosomal sorting complex required for transportation-0
HMW: high molecular weight
Hrs: hepatocyte growth factor-regulated tyrosine kinase substrate
HSE: herpes simplex encephalitis
HSV: herpes simplex virus
IFN: interferon
IL: interleukin
LAMP1: lysosome-associated membrane protein 1
MDA5: melanoma differentiation-associated protein 5
mDCs: myeloid dendritic cells
MoDCs: monocyte-derived dendritic cells
MS: multiple sclerosis
PAMPs: pathogen-associated molecular patterns
PLA: proximity ligation assay
poly(I:C): polyinosinic:polycytidylic acid
PRRs: pattern recognition receptors
RAGE: receptor for advanced glycation end-products
RIG-I: retinoic acid-inducible gene-I
RSV: respiratory syncytial virus
RV: rhinovirus
Syk: spleen tyrosine kinase
TLR3: toll-like receptor 3
TG: trigeminal ganglion
TGN: *trans*-golgi network
TNF: tumor necrosis factor.
