# Supplementary material for: Participation of Endosomes in Toll-Like Receptor 3 Transportation Pathway in Murine Astrocytes
Source: Front Cell Neurosci. 2020 Nov 17;14:544612. doi: 10.3389/fncel.2020.544612 (PMC7705377; doi:10.3389/fncel.2020.544612)
Supplement: Supplementary file 1 [file Table_1.DOCX]

Supplementary Material

# Supplementary Figures


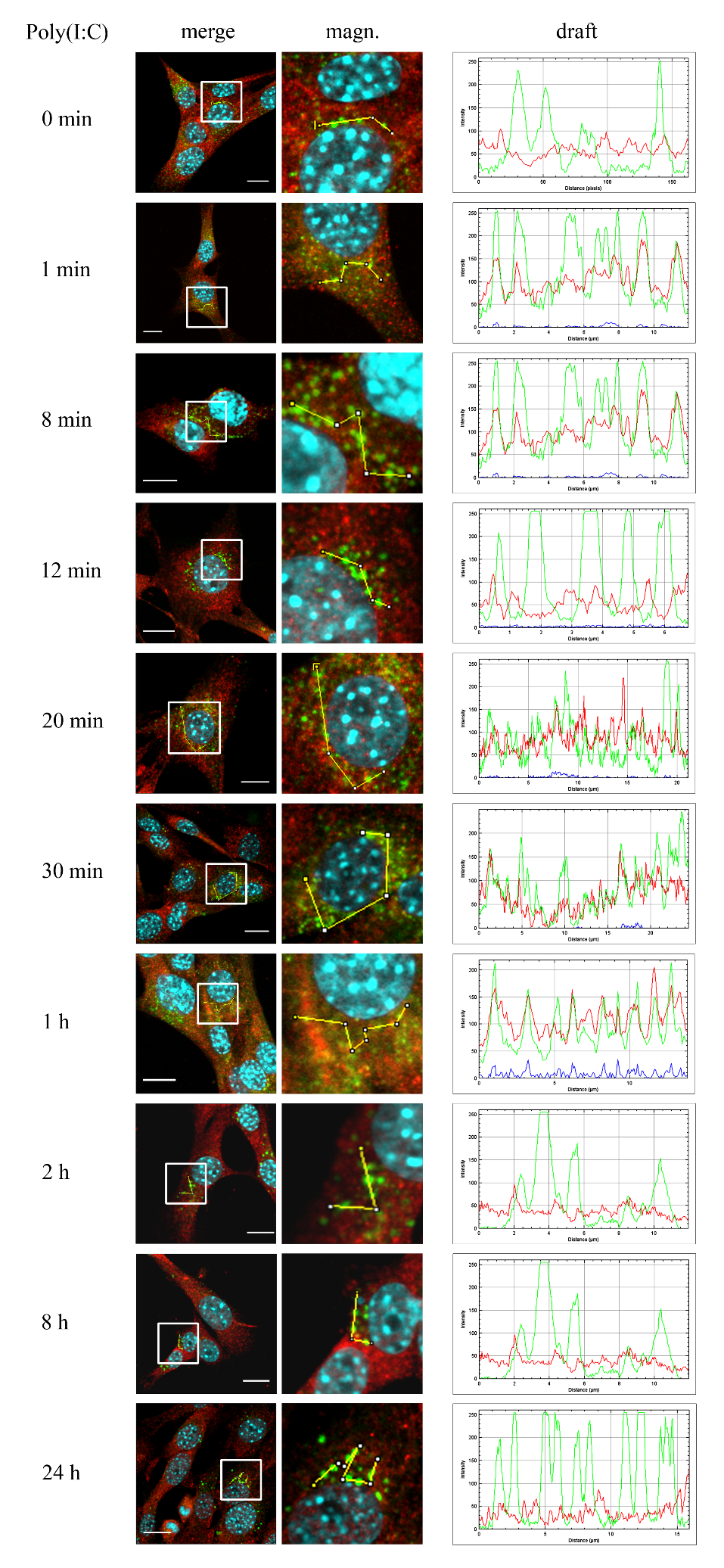


**Supplementary Figure 1.** TLR3 is present in EEA1-labeled endosomes at least up to 1 h following poly(I:C) treatment of C8-D1A cells. Murine astrocytes were not stimulated or stimulated with poly(I:C) for 1 min, 8 min, 12 min, 20 min, 30 min, 1 h, 2 h, 8 h, 24 h. The cells were stained with specific antibodies for EEA1 (green) and TLR3 (red). Magn. – magnification of the selection marked with a white frame on the merged image (merge). The fluorescence intensity of TLR3 (red line) and EEA1 (green line) was measured along the yellow segmented arrows on the enlarged selections (merge). Experiments were performed at least three times and only representative images are shown. Scale bar: 10 µm.


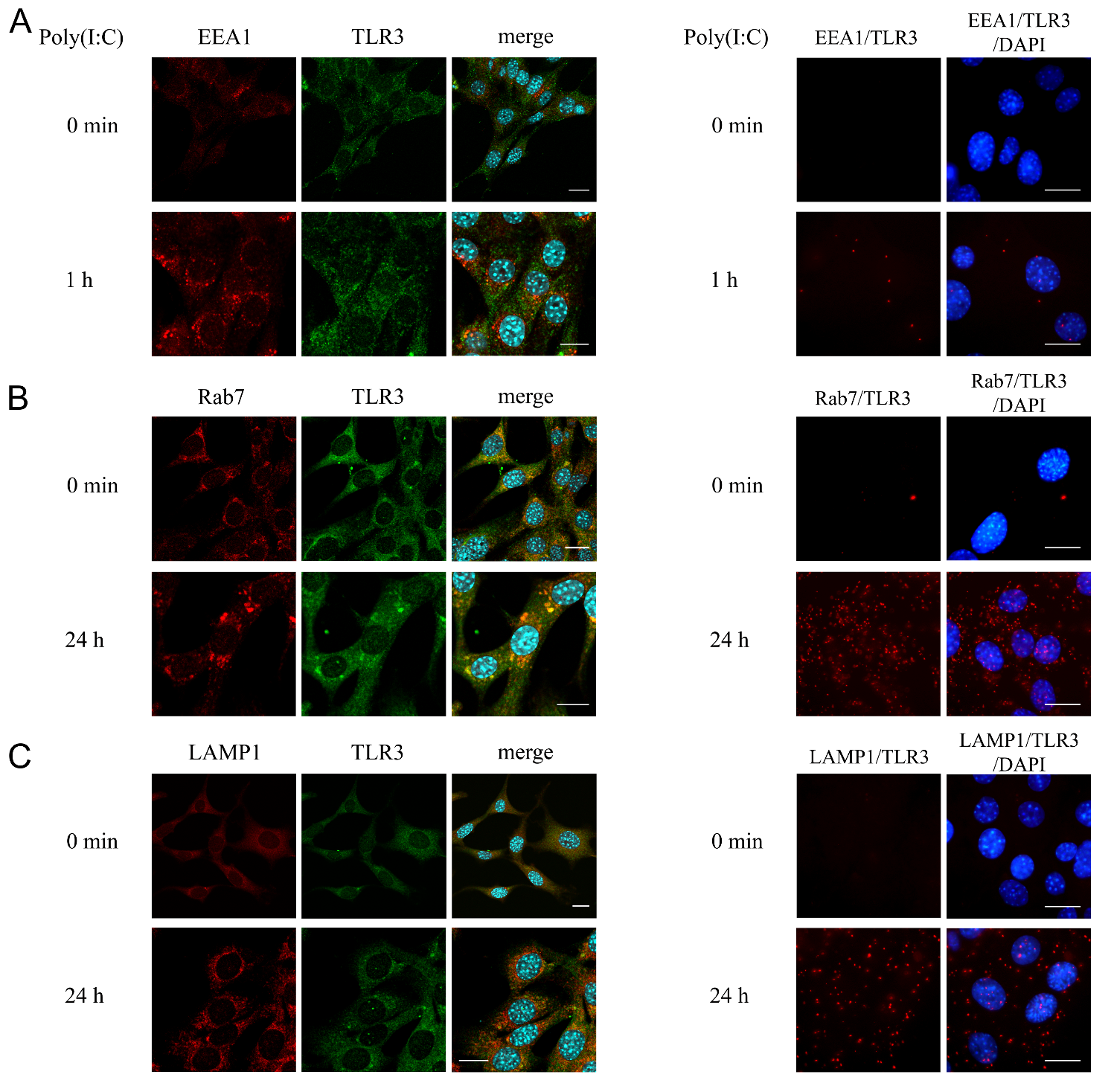


**Supplementary Figure 2.** Astrocytic TLR3 is present in early endosomes, late endosomes or lysosomes following poly(I:C) stimulation of murine astrocytes. C8-D1A cells were not stimulated or stimulated with poly(I:C) for 1 h or 24 h. Co-localization of EEA1 **(A)**, Rab7 **(B)** or LAMP1 **(C)** labeled with rhodamine (red) and TLR3 labeled with FITC (green) occurred in astrocytes treated with the TLR3 ligand for the selected time interval. The interaction of the selected endosomal proteins with TLR3 in untreated cells is also shown. Proximity ligation assays (PLA) of the TLR3 and EEA1 **(A)**, Rab7 **(B)**, or LAMP1 **(C)** interactions were performed in C8-D1A cells stimulated with poly(I:C) for the selected time intervals, and stained with anti-TLR3 antibody and anti-EEA1, anti-Rab7, or anti-LAMP1 antibody, respectively. Signals were detected by the Duolink® *in situ* red starter kit  mouse/rabbit (red). Experiments were performed at least three times and only representative images are shown. Scale bar: 10 µm.


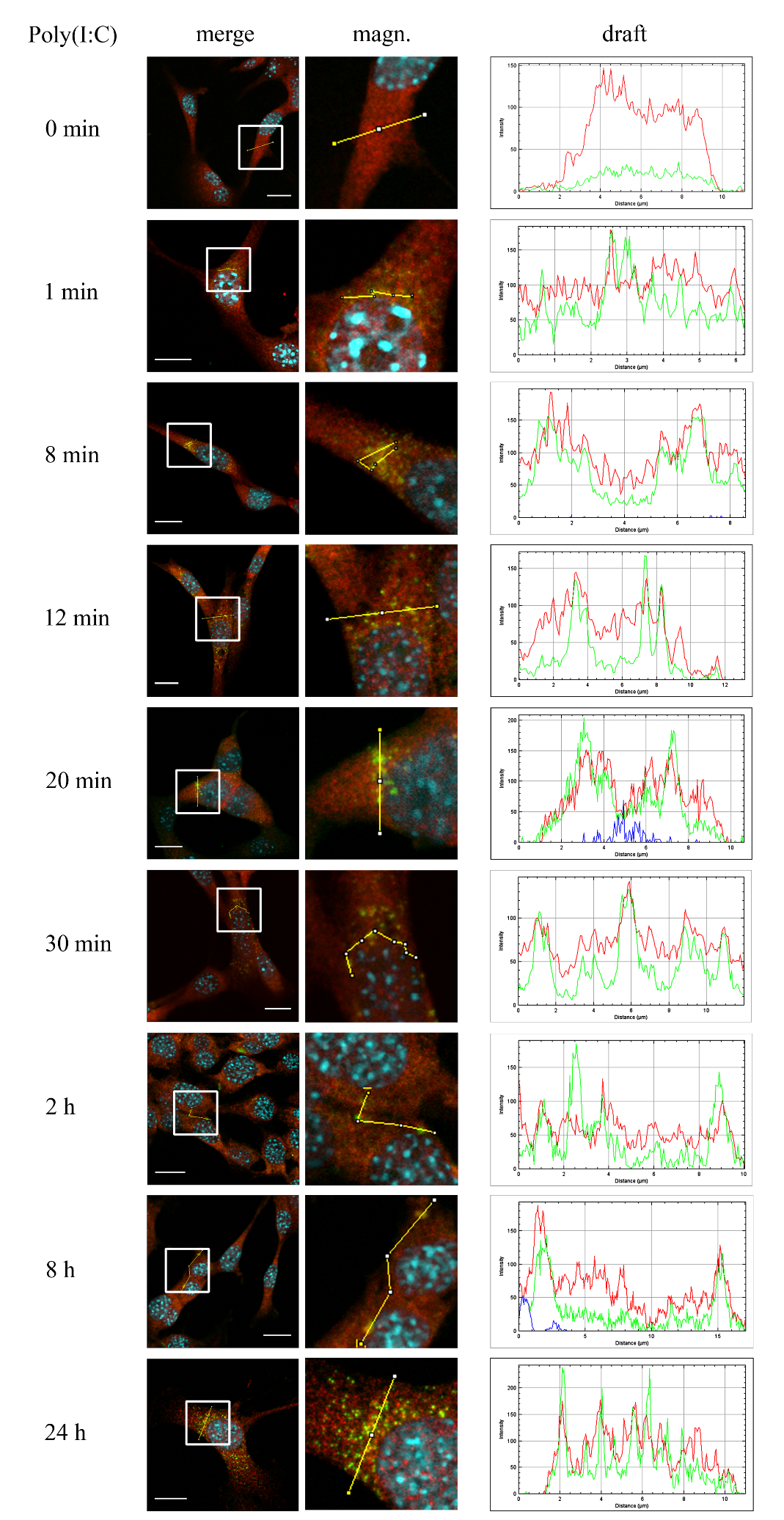


**Supplementary Figure 3.** TLR3 is present LAMP1-labeled endosomes following poly(I:C) stimulation of C8-D1A cells. Murine astrocytes were not treated or treated with poly(I:C) for 1 min, 8 min, 12 min, 20 min, 30 min, 2 h, 8h, 24 h. The cells were stained with specific antibodies for LAMP1 (green) and TLR3 (red). Analysis is based on merged images in Fig. 4. Magn. – magnification of the selection marked with a white frame on the merged image (merge). The fluorescence intensity of TLR3 (red line) and LAMP1 (green line) was measured along the yellow segmented arrows on the enlarged selections (merge). Experiments were performed at least three times and only representative images are shown. Scale bar: 10 µm.
